# Supplementary material for: Splice-Junction-Based Mapping of Alternative Isoforms in the Human Proteome
Source: Cell Rep. Author manuscript; Available in PMC 2020 Jan 15. (PMC6961840; doi:10.1016/j.celrep.2019.11.026)
Supplement: 3 [file NIHMS1546469-supplement-3.zip › DF2/PXD000561/Testis-95-Q9H4G0-VSAMDNTQENSLK.pdf]

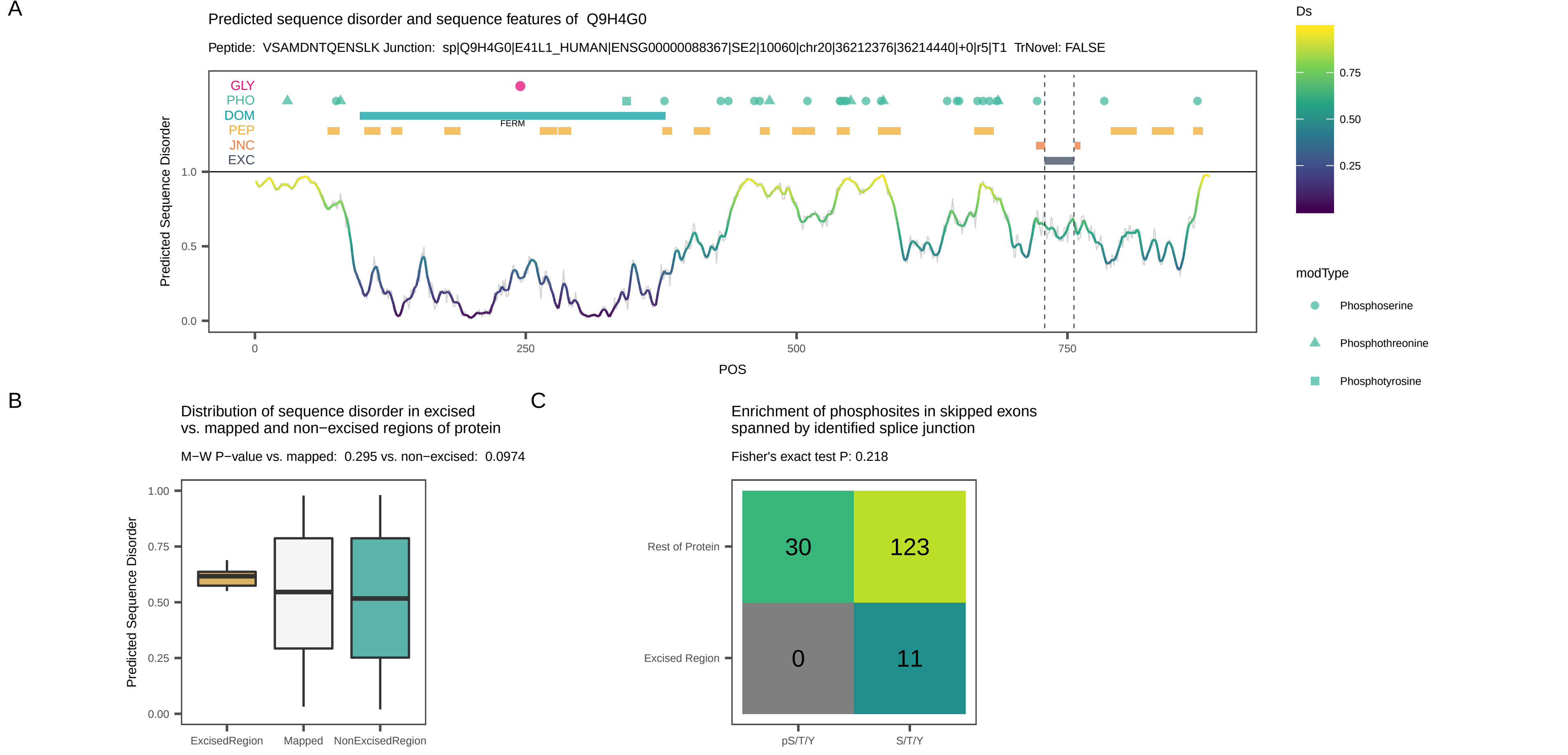

A
Predicted sequence disorder and sequence features of Q9H4G0
Peptide: VSAMDNTQENSLK Junction: sp|Q9H4G0|E41L1_HUMAN|ENSG00000088367|SE2|10060|chr20|36212376|36214440|+0|r5|T1 TrNovel: FALSE
GLY
PHO
DOM
PEP
JNC
EXC
FERM
Predicted Sequence Disorder
1.0
0.5
0.0
0
250
500
750
POS
Ds
0.75
0.50
0.25
modType
Phosphoserine
Phosphothreonine
Phosphotyrosine
B
Distribution of sequence disorder in excised
vs. mapped and non−excised regions of protein
M−W P−value vs. mapped: 0.295 vs. non−excised: 0.0974
Predicted Sequence Disorder
1.00
0.75
0.50
0.25
0.00
ExcisedRegion
Mapped
NonExcisedRegion
C
Enrichment of phosphosites in skipped exons
spanned by identified splice junction
Fisher's exact test P: 0.218
Rest of Protein
Excised Region
30
123
0
11
pS/T/Y
S/T/Y
